# Influence of Different Bonding Agents and Composite Resins on Fracture Resistance of Reattached Incisal Tooth Fragment

**Published:** 2014-03

**Authors:** AR. Davari, M. Sadeghi

**Affiliations:** a Dept. of Restorative Dentistry, School of Dentistry, Shahid Sadoughi University of Medical Sciences, Yazd, Iran.; b Dept. of Restorative Dentistry, School of Dentistry, Rafsanjan University of Medical Sciences, Rafsanjan, Iran.

**Keywords:** Reattachment technique, Tooth fracture, Dentin bonding agent, Composite resin

## Abstract

**Statement of Problem:** Reattachment of the fractured tooth fragment should be considered as a conservative treatment and valid alternative to a composite restoration.

**Purpose:** This in vitro study was to evaluate the influence of different adhesives and composite resins on fracture resistance of dental fragment reattached to the sectioned incisal edges.

**Materials and Method:** 120 sound human maxillary central incisors were selected under standard conditions and randomly divided into 3 groups, 12 sound teeth were used as a control group and the remaining teeth were assigned to 3 groups (n=36) and each group into three subgroups (n=12). The incisal third of the samples was sectioned using a diamond disk and the respective fragments were then reattached utilizing different intermediate restorative materials, namely: i) adhesive materials alone (OptiBond S or OptiBond XTR or OptiBond All-in-One; ii) Premise flowable composite and iii) Point 4 composite in the one of the mentioned adhesive interface. After storage for two weeks at 37°C and 100% humidity and then thermocycling; shear bond strength (SBS) was recorded in kilogram force (kgf) by applying a load in the middle incisal third with a Zwick Universal Testing Machine at a cross-head speed of 1 mm/min. Data was analyzed with one-way ANOVA and Tukey HSD (p< 0.05).

**Results:** The control group had a significantly higher SBS than other groups (p= 0.001); the highest SBS values were obtained using the Premise flowable composite and OptiBond S adhesive (112.44±30.46 MPa); and the lowest with OptiBond All-in-One alone (33.97± 15.63 MPa).

**Conclusion:** Although, none of the tested materials provided fracture resistance similar to that found with the intact maxillary central incisors; utilizing the Premise flowable composite and OptiBond S adhesive improved the SBS of the reattached fragment than other materials.

## Introduction

Coronal fractures of the anterior teeth are the most frequent form of traumatic dental injury that mainly affects children and adolescents [1-3]. On average, 1 in 4 people will suffer a crown fracture involving mainly the maxillary central incisors [3-6]. The traditional conservative treatment of crown fractures has been the restoration of involved teeth with composite resin and dental bonding systems [7-8].

Despite the recent developments in adhesive materials and restorative techniques, there is no restorative material that can reproduce the esthetic, functional needs and the natural dental structures [9]. Therefore, when the fractured fragment is available and ample enough to be used after dental fracture [10-11], reattachment should be considered the treatment of choice as the most conservative treatment approach [11-12]. Reattachment of coronal fragments is an imperative technique for restoring fractured teeth that provide advantages over composite resin restorations or full-coverage crown [11, 13]. It is a simple, less time-consuming and low-cost method. It allows the maintenance of incisal function in dental structure and provides good and long lasting esthetics [10-11, 13]. The method maintains the natural characteristics of wear, shape, surface texture and color and produces minimal tooth loss [10, 14]. Consequently, it improves function and provides positive emotional response from the patient [10-11].

From a clinical standpoint, this technique promises the minimal intervention concept [15]. Since this method is simple and conservative and provides the good fragment retention and satisfactory esthetics, the re-attachment of the coronal fragment seems to be a practical alternative to placement of conventional composite resin restorations in the management of fractured anterior teeth. It also guarantees a complete restitution in integrum of the tooth [7, 16].

The development of resin-based materials that offer high bond strength values has made it possible to reattach the fragments by employing the modern dentin bonding agents or adhesive luting systems without imposing an additional retentive preparation of the tooth or fragment [7-8, 17-18]. Some researchers have investigated the reattachment using bonding agents alone [8, 17, 19-21], or bonding agents with flowable resins, dual cured, self-cured or light-cured luting cements. They have reported that reattachment using these materials may achieve functional and esthetic success [7-8, 18, 22-24].

Reis et al. [21] reported that a simple reattachment with no further preparation of the fragment or tooth was able to restore only 37.1% of the intact tooth’s fracture resistance. In their study, a buccal chamfer recovered 60.6% of fracture resistance and bonding with an over contour and the placement of an internal groove nearly restored the intact tooth fracture strength with recovering values of 97.2 and 90.5% respectively. They advocated the necessity of using additional preparations to enhance the retention of the reattached fragment.

In general, these findings highlight the need for further investigations regarding the effects of new adhesive materials that are being continuously made and introduced for clinical use. Therefore, the purpose of this study was to evaluate and compare the shear bond strengths of fractured human maxillary central incisors’ fragments reattached with different composite resins and adhesive resin materials. The null hypotheses were considered as there is no difference between the effectiveness of three adhesive materials and between the microhybrid and flowable composite resins, employed as an intermediate layer, on the shear bond strength (SBS) of reattached fragments.

## Materials and Method

A total of 120 sound human maxillary central incisors, extracted for therapeutic reasons, were used in this study. The teeth were cleaned from the soft tissues and calculus with curettes and ultrasonic devices and kept in an aqueous buffered solution of formaldehyde (Yekta Chem Co.; Tehran, Iran) for two hours to be decontaminated. The teeth were inspected under optical magnification (×4) [16] to rule out the presence of cracks, caries or any other kind of structural defects and then stored in a sterile saline solution at room temperature until prepared for a maximum period of three months. The teeth were randomly assigned to three different treatment groups (n=36) and then each treatment group into three subgroups (n=12). A total of 12 specimens, without any preparation or treatment, were assigned as the control group.

The crown of teeth was measured on the labial side, from the cervical line to the incisal edge and from the mesial surface to the distal surface by using a digital caliper. This measurement was then divided into transversal and longitudinal thirds by a marker. Each tooth was embedded in an auto polymerizing acrylic resin (Simplex Rapid; *KemDent***,** Wiltshire, UK) at cemento-enamel junction. The mark line at the junction of the incisal and medal third in the treatment groups were sectioned perpendicularly to the long axis of the tooth with a diamond disk (D&Z Diamant GmbH; Lemgo, Germany) using a low-speed handpiece under cooling water; thus, for each tooth, one fragment was obtained.

The treatment groups were reattached as follows:

Group 1 (Subgroups 1-3): The reattachment technique was performed with the adhesives OptiBond S (two-step etch-and-rinse, Fifth-Generation), OptiBond XTR (two-step self-etch, sixth-Generation) and OptiBond All-in-One (one-step self-etch, seventh generation) (Kerr Corporation; Orange, CA, USA) according to manufacturer’s instructions respectively (Table 1). The adhesive resins were applied to both surfaces without light curing to prevent misfit of the bonded parts. The fragments were then reattached to the remaining tooth and light cured for 10 seconds using a light-emitting diode (LED) light curing unit (Demetron A.2; Kerr Italia, S.p.A., Scafati, Italy) on both labial and lingual surfaces with a light intensity of 1,000 mW/cm2, while pressing the coronal fragment against the matching tooth part.

Group 2 (Subgroups 4-6): After applying the adhesive systems to both surfaces as in group 1 (subgroups 1-3), a coat of nano-filled (Premise Flowable; Kerr Corporation, Orange, CA, USA) flowable composite was applied to the tooth and the fragment. The fragment was reattached to the remaining tooth using hand pressure and the excess material on both labial and lingual surfaces was removed using a micro brush and light-cured for 20 seconds on both labial and lingual surfaces.

Group 3 (Subgroups 7-9): The treatment was the same as group 2 (Subgroups 4-6) but a microhybrid composite resin (Point 4, Kerr Co, A3 Body Shade) was used instead of flowable resin.

Group 4 (control group): No preparations or treatments were performed in this group.

To avoid drying and cracking during the laboratory procedures, the samples were kept wet. After reattachment procedures, the specimens were stored in distilled water at 37°C and 100% humidity for two weeks and then were thermocycled for 1500 cycles between 5°C and 55°C at a settle time of 30 s. The shear bond test was performed using a universal testing machine (*Zwick* GmbH & Co.; Ulm, Germany) at a cross-head speed of 1 mm/min in a compression mode. The specimens were fixed at 90 degrees to the load applied and a metal cone with 1.0mm tip cut vertically to the center of the incisal third of the labial surface to simulate real clinical situations as possible. The maximum load to failure was recorded for each specimen and the shear bond strength was recorded in kilogram force (kgf).

The statistical analysis was performed with SPSS-18 (SPSS Inc.; Chicago, IL, USA). The Kolmogorov–Smirnov test was applied to verify the normal distribution of data. One-way ANOVA and Tukey HSD tests were used to determine the differences among groups and subgroups. The level of significance for all tests was set to 0.05. 

**Table 1:** Various adhesive materials used in the study and mode of their applications according to the manufacturers' instructions

**Material Name**	**Manufactures’ Instructions**
OptiBond S (Two-step etch-and-rinse)	1. Etch enamel and dentin for 15s with 37.5% phosphoric acid (Kerr Gel Etchant; Kerr Co) 2. Rinse thoroughly, ensure that all etch is removed 3. Dry lightly (do not desiccate) 3. Apply the adhesive and rub for 15s 4. Air thin for 3s 5. Light cure for 10s 6. Place composite and light cure for 20s
OptiBond XTR (Two-step self-etch)	1. Apply the self-etch primer using a micro brush with a scrubbing motion for 20s 2. Air thinning for 5s using medium pressure 3. Shake the adhesive briefly 4. Apply the adhesive using a light brushing motion for 15s 4. Air thinning using medium to strong pressure for at least 5s 5. Light cure for 10s 6. Place composite and light cure for 20s
OptiBond All-in-One (One-step self-etch)	1. Shake the bottle for 10s 2. Apply the adhesive and rub for 20s 3. Apply a second layer of adhesive in the same fashion 4. Air thinning lightly for 5s 5. Light cure for 10s 6. Place composite and light cure for 20s

## Results

The data normality was confirmed by using the Kolmogorov-Smirnov test and the one-way ANOVA revealed a significant difference between the experimental groups (p= 0.001). The Tukey HSD test showed that the control group had a significantly higher SBS compared to all the other groups (p= 0.001). The means and standard deviations of SBS values of the test groups are pre-sented in table 2.

**Table 2:** The means and standard deviation of SBS values (Kgf) and recovering rate of SBS in relation to control group for the tested groups. (112.44 ± 30.46 MPa) (33.97 ± 15.63 MPa)

**Groups**	**Subgroups**	**Mean ± SD**	**Recovery (%)**
I	OptiBond S alone	55.24 ± 14.89	17.43
	OptiBond XTR alone	47.05 ±19.21	15.51
	OptiBond All-in-One alone	33.97 ± 15.63	11.49
II	Premise Flowable +OptiBond S	112.44± 30.46	35.41
	Premise Flowable +OptiBond XTR	90.11 ± 26.53	29.50
	Premise Flowable +OptiBond All-in-One	74.59 ± 13.01	24.37
III	Point 4 Composite + OptiBond S	69.65 ±16.65	22.29
	Point 4 Composite + OptiBond XTR	74.29 ± 10.39	23.48
	Point 4 Composite + OptiBond All-in-One	44.85 ± 11.03	14.22
VI	Control	332.86 ± 89.16	100

Based on the results of the present study, the highest SBS values were observed in the samples reattached with the Premise flowable composite and OptiBond S adhesive; and the lowest with the OptiBond XTR adhesive alone.

The use of Premise flowable composite, in the adhesive interface, significantly increased the SBS values compared to those reattached with Point 4 composite in the adhesive interface (p= 0.003) and to those with the adhesives alone (p= 0.001). There was no statistically significant difference between the samples reattached with the Point 4 composite in the adhesives interface and adhesives alone (p= 0146).

There was statistically significant difference among the samples reattached with the adhesives alone compared. Among them (p= 0.013). While, the OptiBond S displayed significantly higher SBS values than the group OptiBond All-in-One (p= 0.01), but there was no statistically significant difference between the Opti Bond XTR with OptiBond S (p= 0.460) and OptiBond All-in-One (p= 0.149).

When the Premise flowable composite in conjunction with the adhesives was used to reattach the fragments, the Premise flowable composite and OptiBond S revealed significantly higher SBS values than the Premise flowable composite and OptiBond All-in-One (p= 0.002), but there was no statistically significant difference between the Premise flowable composite and OptiBond XTR with Premise flowable composite and OptiBond S (p= 0.08) and Premise flowable composite and OptiBond All-in-One (p= 0.281). Nevertheless, the difference among the three adhesives was significant (p= 0.002). 

When the fragments were attached with the Point 4 composite and the adhesive systems, the Point 4 composite and OptiBond All-in-One had significantly lower SBS than Point 4 composite and OptiBond S and Point 4 composite and OptiBond XTR (p= 0.001), but the difference between the Point 4 composite and Opti-Bond S; and Point 4 composite and OptiBond XTR was not significant (p= 0.659). The samples reattached with the Point 4 composite in the adhesive interface showed slightly higher SBS than those reattached with the adhesives only, however, this difference was not significant (p= 0.146).

When the subgroups were compared with each other, only the samples were attached with the Premise flowable composite and OptiBond S had significantly higher SBS than those which were attached with the OptiBond S alone (p= 0.002), but the difference between the other counterpart subgroups was not significant (p> 0.05).

**Figure 1:**
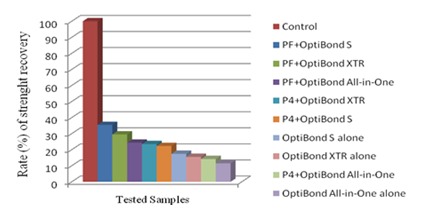
Flowable = PF; Point 4 = P4; Recovering rate of SBS (kgf) in relation to control group for testing groups.

The highest and lowest SBS recovery values were obtained when the fragments were reattached with the Premise flowable composite and OptiBond S (35.4% of control); and OptiBond All-in-One alone (11.5% of control), respectively (Figure 1).

## Discussion

In the present study, the human maxillary central incisor teeth were used to perform the tests since these teeth fracture [1-3]. In addition, the specimens used for the experiment were teeth extracted for periodontal reasons, which are usually teeth of older people, whereas trauma happens usually in younger patients. Aging can cause alterations, especially in dentin, which can decrease the retention force of restorations [25]. However, all teeth were extracted from patients of similar ages, providing a degree of homogeneity which avoids potential disparities [26].

In this study, the incisal edge of specimens was sectioned using a diamond disk. This is the limitation of our study that the fractures differ from the natural fractures and the fragment probably will not fit well over the remaining tooth structure as precisely as in the case of a natural fracture. This situation could make it difficult to use an adhesive as a unique reattachment agent since a thicker layer of material may be necessary to fill the gaps present in the interface. 

Cutting with a bur produces a smear layer which is otherwise, not present on a fractured surface. A fractured surface often runs parallel to the main direction of the enamel prisms, while the orientation of the sectioned surface is dictated by the direction of the cut. The sectioned fragment establishes standardized and repeatable condition that is absolutely necessary for an in vitro study [12, 16, 19-21, 27].

Also, the method employed to obtain the fragment of the incisor margin was to provoke its fracture and not to cut the crown of the teeth [28]. Loguercio et al. [27] evaluated the effect of fractured or sectioned fragments on the fracture strength recovery of different reattachment techniques. They concluded that no differences could be detected among reattachment techniques when fragments were obtained by sectioning; but the force necessary to cause the detachment of the sectioned fragment was significantly lower than the force recorded in fractured teeth.

Furthermore, in the group with cut teeth, the percentage of resistance of the various re-bonding techniques did not demonstrate any significant differences, unlike what happened in the group with fractured teeth [29]. The dentinal bonding systems show different characteristics of adhesion to the enamel and to the dentin for which it is necessary to be very careful when preparing equivalent bonding surfaces [28]. In the attempt to obtain an equal amount of area exposed, all of the teeth were cut at the same distance from the incisor margin i.e. incisal third.

The current study tried to incredibly reduce the variation in resistance to fracture which results from the thickness of the layers of enamel and dentin, however, the anatomy of the surface produced by the cutting is certainly different from the surface resulted from the natural fracture. 

The findings of this study showed that none of composite resins and adhesive materials was able to attain the resistance against shear stresses as much as the intact teeth. The fracture resistance recovery values were between 11.5-35.5% of intact teeth, which is in accordance with previous findings in the literature [17, 19-21, 27]. The results of the present study rejected the both null hypotheses, as there was a significant difference in the fracture resistance of reattached teeth among the tested adhesives and between the tested composite resins.

When the adhesives were used alone for reattachment of fragments, the etch-and-rinse adhesive OptiBond S yielded the highest shear strength value than the one-step self-etch adhesive; OptiBond All-in-One. However, it was similar to that of the two-step self-etch adhesive, OptiBond XTR. In general, the reattached teeth with the tested adhesives alone had the lowest SBS. An in vitro study also concluded that the worst fracture resistance and the lowest failure load were obtained from the bonded specimens with adhesive alone [19]. Conversely, Farik et al. [23] indicated that the fracture resistance using dentine bonding agents alone, without any additional tooth preparation, is the same as that those obtained with intact teeth. Moreover, Reis et al. [21] concluded that the use of the adhesive system alone, or in combination with other materials such as flowable resins and filled resins, gives similar results when the fragment is reattached without additional preparations.

The development of adhesive systems that are always becoming more efficient has encouraged many authors to employ only these systems for reattachment of the fractured fragments [8, 17]. Despite of the ever-increasing popularity of self-etching bonding agents, adhesive systems that employ phosphoric acid as a separate conditioner still represent the gold standard of reliable and strong enamel bonding [8, 30]. Nevertheless, self-etching adhesives can provide dentin bond strengths that are equal to or greater than those achieved by etch-and rinse adhesives [30], whereas many in vitro studies have discouraged the use of these materials on intact enamel because of significantly lower bond strengths, greater microleakage and shallow etching patterns that prevent good penetration of the bonding resin [31-32].

Accordingly, Pusman et al. [12] suggested that self-etching adhesives to be applied to the fractured surfaces following selective phosphoric acid etching of the enamel surfaces and this procedure had different effects on the fracture strength recovery values obtained by the self-etch adhesives. On the other hand, selective phosphoric acid etching of enamel could increase the bond strengths of some single-step self-etching adhesives to the levels that were comparable with or greater than those of etch-and-rinse adhesive systems [33-34].

The ultra-morphological findings demonstrated that the existence of voids and microcracks along the fragment–tooth adhesive interface could limit the efficiency of such clinical procedures [12]. Especially, the microcracks could act as notches that induce further crack propagation under intermittent mechanical loading in vivo; and possibly lead to the failure of reattached fragments because of subcritical cracking [35-36]. The voids may also weaken the integrity of the tooth- adhesive interface [12].

Based on the present findings, fracture resistance produced for specimens with Premise flowable composite in the adhesive interface generated the highest fracture resistance recovery, which is in accordance with previous findings in literature [[8, 22, 24].

Nevertheless, this bond strength value was about 35% of that produced in intact teeth. Therefore, this value cannot guarantee an excellent clinical performance. The amount of strength recovery needed to keep the fragment in position long-term still remains unknown [12]. Perhaps fracture strengths that are as low as 50–60% might be sufficient if these values confirmed by clinical studies [10]. The flowable composites flow adequately in the reattachment site [37]; therefore, making good apposition between the fragment and the tooth, particularly when there is a small loss of dental structure after coronal fracture. Despite this fact, improved fracture resistance was not observed using a flowable resin for re-attachment when compared to a hybrid composite resin [21].

Again, when the adhesives were used together with the Point 4 composite for reattachment, OptiBond XTR and OptiBond S displayed the best fracture strength; whereas the relatively inferior fracture strength recovery value of OptiBond All-in-One was comparable with those obtained both two-step self-etch and etch-and-rinse adhesive test materials. Demarco et al. [19] concluded that the light-cured composite recovered 20% of the fracture resistance of intact bovine incisors, when applied without preparation.

Point 4 composite is a light-cured, resin-based composite dental restorative that contains approximately 77% by weight (59% by volume) inorganic filler with an average particle size of 0.4 microns [38]. The composite resins provide better mechanical properties and thus could reinforce the re-attachment interface [17]. Nevertheless, because the light-cured composite resin is heavy-filled, they may not flow adequately in the re-attachment site [37], particularly when there is a small loss of dental structure after coronal fracture. In addition, the presence of composite in the reattachment interface may impair the long-term aesthetic since the color of composite resins change with aging [19, 39].

Fracture resistance is typically reported as strength (force /area, MPa) [40]. Since it was difficult to determine the area of the bonded surface, the results are reported as a fracture force (kgf) in this study. Also, to obtain standard bonding areas, the specimens were cut down to equal dimensions. There has been no report on the standardization of bonding areas of reattached teeth for shear bond strength measurements; therefore, careful attention was paid on preparing standard bonding areas in the teeth. This was an attempt to reduce the variation in the bond strength results that reported in previous studies using similar methodologies [19, 26-28]. The differences between results of previous studies and the present study may be related to the various methodologies used to perform the tests, including the tested materials, loading speed and thermal cycling [12, 19, 28, 35].

Recently, Singhal et al. [41] reported that the fracture resistance of reattached teeth, using resin modified glass ionomer cement, compomer; composite resin and dual curing resin cement varied 24-51% of that for an intact tooth. Reattachment with composite resin and the resin-modified glass ionomer cement provided the highest and the weakest fracture resistance respectively. As Andreasen et al. [17] suggested the reattachment technique is a realistic alternative to composite resin build up, although only half of the strength of intact teeth is achieved. Also Demarco et al. [19] stated in such a situation, the patient should be cautioned to limit the function of the fractured teeth. Nevertheless, it is reported that utilizing Panavia F cement and polyethylene fiber (Ribbond) using an internal groove technique gives additional strength to the reattached tooth fragment equal to an intact tooth [42]. Based on the present study, the fracture resistance reattached fragments with different materials was not able to restore the fracture resistance of the intact teeth. Thus, the investigation of new materials and techniques is necessary to improve the resistance and the longevity of reattachment of coronal fragments.

## Conclusion

In light of the results obtained by this in vitro study and within its limitations, it is possible to conclude that:

1. None of the tested materials provided fracture resistance similar to that found with the intact maxillary central incisors, leading to reject the null hypothesis.
2. The best fracture resistance was obtained when the fragments were reattached with an intermediate Premise flowable composite layer and it was able to recover the fracture resistance just up to 35.5% of intact teeth.
3. The worst fracture resistance was obtained when the samples were bonded with adhesive materials alone.

The current research was an in vitro study; therefore, further studies are required to elucidate the effect of these materials on fracture resistance.
